# Design and Construct Validity of a Postural Control Test for Pre-Term Infants

**DOI:** 10.3390/diagnostics13010096

**Published:** 2022-12-29

**Authors:** Katarzyna Kniaziew-Gomoluch, Andrzej Szopa, Zenon Kidoń, Andrzej Siwiec, Małgorzata Domagalska-Szopa

**Affiliations:** 1 Department of Physiotherapy, Medical University of Silesia in Katowice, 40-752 Katowice, Poland; 2 Neuromed, Rehabilitation and Medical Center, 40-698 Katowice, Poland; 3 Department of Electronics, Electrical Engineering and Microelectronics, Silesian University of Technology, 44-100 Gliwice, Poland; 4 John Paul II Pediatric Center, 41-218 Sosnowiec, Poland; 5 Department of Developmental Age Physiotherapy, Medical University of Silesia in Katowice, 40-752 Katowice, Poland

**Keywords:** general movements, posturometic test, pre-term infants, postural control, force platform

## Abstract

A review of the literature indicated that the greatest prognostic value for predicting motor impairment in high-risk infants is the absence of fidgety movements (FMs) at 3 months of post-term age. The purpose of the present study was to characterize a new posturometric test (PT) based on a center-of-pressure (CoP) movement analysis, in terms of design and construct validity, for the detection of postural control disturbances in pre-term infants. The comparative studies were carried out between pre-term infants who presented normal FMs (18 participants) and infants with absent FMs (19 participants), which consisted of the analysis of the CoP trajectory and CoP area in supine and prone positions using the force platform. New PT was performed simultaneously with GMs recorded using a force platform. Statistical analyses revealed significant differences between the groups of infants who presented absent FMs and normal FMs for almost all CoP parameters describing spontaneous sway in the supine position. Based on these preliminary results, it can be concluded, that the application of PT based on the analysis of CoP trajectory, area, and velocity in the supine position has been demonstrated to be valid for the detection of postural control disturbances in pre-term infants.

## 1. Introduction

Premature infants are born at a gestational age ranging from 22 to 37 weeks, with a body weight of up to 2500 g [1]. Recent studies have reported that pre-term infants are more prone to neurodevelopmental impairment (NDI) than full-term infants [1,2,3]. Moreover, there is evidence that the occurrence of cerebral palsy (CP) is associated with pre-term birth and extremely low birth weight in infants [4,5,6]. The prediction of NDI (including CP) in pre-term infants is challenging, as it requires the use of diagnostic methods and tools to recognize early symptoms (occurring during the first 3 months of life) that would indicate later development of NDI [7]. 

The results of the review of the literature showed that Prechtl’s general movements assessment (GMA) is currently the most evidence-based clinical approach for the prediction of motor impairment in high-risk infants [7,8]. 

GMA is a standardized functional assessment based on the subjective visual assessment of young infants’ general movement (GM) patterns using the Gestalt perception phenomenon, whose advantages are both the ability to perceive complex entire patterns or configurations and sensitivity to individual components [9,10,11,12]. 

The presence of normal GMs, especially fidgety movements (FMs) at 3 to 5 months after term, especially if they co-occur with other smooth and fluent movements, are very likely to show neurologically normal development [11,12]. Conversely, the presence of abnormal GMs, in general, indicate an increased risk for later neurological dysfunction [9,10]. While poor repertoire GM patterns [PR] are not early markers of adverse neurological outcomes, the consistent cramped-synchronized GMs patterns [CS] at pre-term and term age, and the absence of FMs at post-term age, are early signs of an atypical outcome, mainly CP [12,13,14].

GMA presents high reliability for predicting CP in high-risk infants, with a reported sensitivity of 98% (95% confidence interval, CI 74–100%) and specificity of 91% (95% CI 83–93%) [15,16]. Even though GMA currently presents the highest value for predicting NDI, it is based on a subjective visual assessment. 

With advancing computer science, multiple systems based on sensor technology have been adapted for the identification of GMs in young infants [17,18]. There are several study articles on monitoring the body movements of infants based on either direct sensing using body-worn miniaturized movement sensors [19], indirect sensing based on visual sensors (2D or 3D video) [20,21,22,23], or both [24,25]. While the obtained results of GMA based on motion sensors appear very promising, none are sensitive enough to determine such movement parameters that are able to distinguish normal from abnormal GMs [17,18,19,26]. Moreover, according to the results of a review of “automated movement recognition technologies to assess infant movement” performed by Marcroft et al., the applicability of these techniques is limited by difficulties attaching special markers to the very small limbs of infants [27]. 

In recent years, center-of-pressure (CoP) data have been used for the analysis of infant movements [28,29]. COP is defined as the point where the ground reaction force is applied, and it represents the center point of the entire pressure of the body in the ground contact surface, and the analysis of CoP trajectory and CoP area are common in the study of human postural control [30]. Although, the CoP analysis of these studies was sufficient to screen the infants grouped by birth status (pre-term/term) [28,29] these studies did not relate to any observational clinical classification of high risk for CP according to the GM concept. 

Although, several studies compared various CoP metrics between infants grouped by future motor outcome (typically developing infants vs. infants with or at risk for CP), using force plates, they were used in infants over one year and assessed sitting postural control [31,32]. 

To the best of our knowledge, the validation of CoP in young infants (up to 3 months) grouped by future motor outcome (typical/impaired postural control) and measured using a force platform in a horizontal (supine or prone) position has not been published thus far. 

Taking into consideration the close relationship between postural control and movement [33], it can be hypothesized that abnormal GMs are accompanied by atypical postural control. Thus, the current study attempted to identify typical vs. impaired postural control in pre-term infants in horizontal positions (i.e., supine and prone) using the force platform. Using clinical GMA as a reference, the present study aimed to characterize a new posturometric test based on CoP movement analysis in terms of design and construct validity for the detection of postural control disturbances in pre-term infants. For this purpose, comparative studies were carried out between pre-term infants who presented normal FMs (indicating normal future motor outcomes) vs. absent FMs (indicating later neurological dysfunction). 

## 2. Materials and Methods

This study was approved by the Bioethical Committee of the Medical University of Silesia in Katowice under resolution No. KNW/0022/KB1/148/14, and informed consent was obtained from all participating families. It also conforms to the Helsinki Declaration. All of the infant’s parents provided written informed consent before the study, including enrolment and data collection. 

### 2.1. Participants

**Figure 1:**
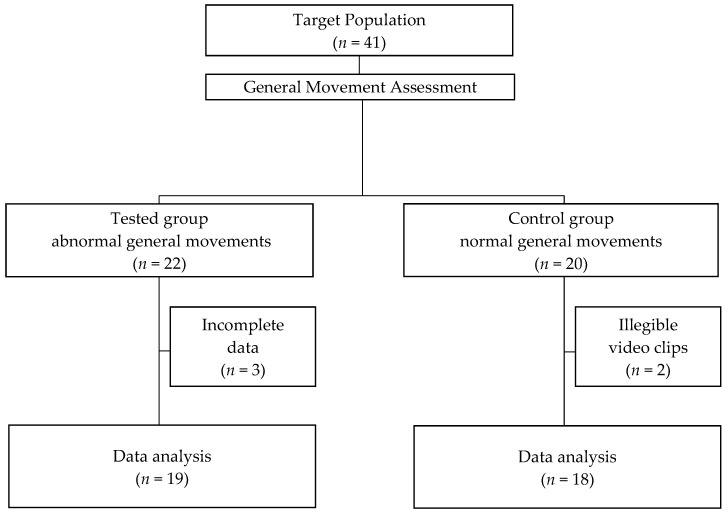
Study population.

Twenty-two pre-term infants with a high risk of developing CP, who were cared for by the local Neonatal Counsel Clinic of Public Clinical Hospital, were enrolled in the study. The inclusion criteria for the study group were as follows: (1) infants’ gestational age at birth was between 25 and 33 completed weeks; (2) brain ultrasound abnormalities were present; and (3) the presence of absent FMs had been confirmed by GMA. Of the 22 infants enrolled in the study group, 3 infants were excluded from the study due to incomplete data. The target population in the study group included 19 pre-term infants (6 girls and 13 boys), who presented absent FMs at 3–5 months after term (Figure 1). The most frequent ultrasound abnormalities in this group of infants included intraventricular hemorrhage (IVH grade 3 and 4), periventricular leukomalacia (PVL) grade 3 (extensive periventricular cystic lesions) and grade 4 (extensive cystic lesions in the deep white matter), as well as periventricular venous infarction and post-hemorrhagic ventricular dilation. 

A group of 20 individuals with (1) normal brain ultrasound findings and (2) normal FMs at 12–14 weeks after term, matched for sex and age (in a 1:1 case-control manner) to the children from the study group, were enrolled sequentially in the study as a control group. As the video clips of two participants were illegible, it was decided to omit them in the final analysis; therefore, 18 infants (6 girls and 12 boys) were included in the study analyses (Figure 1).

Participation was excluded if: (1) they had major congenital anomalies and genetic syndromes; (2) they were clinically unstable; (3) they had an infection or inflammation during the examination; or (4) they were newborns whose parents/legal guardians had not approved the examination. The demographic and clinical characteristics of the participants are presented in Table 1.

### 2.2. Examinations

Each examination consisted of the videotaping of GMs, GMA, and PT. Recordings of GMs consisted of videoing infants’ spontaneous activity twice: first, at term birth (i.e., at 40 postmenstrual weeks), and second, at 12–14 weeks after the infant’s due date—according to the standard methodological principles of Prechtl’s method of GMA [9,10,13]. 

During recording, the infant was placed in a supine position on a force platform, dressed in clothes not restricting movement or—in the case of an infant lying in an incubator—only in a diaper (no pacifier or toys). The temperature of the room was comfortable for the infants. A digital camera was placed on a tripod approximately one meter above the incubator or examination table. The frame included the entire infant, including the face. Recordings were made over 1 h after a meal and when the infant was as active as possible, and each video was recorded for an hour. Infants were only recorded if they were not sleeping or crying; otherwise, videotaping was interrupted and postponed. 

#### 2.2.1. GMs Assessment

All videos were analyzed (offline) by three independent observers (knowing postmenstrual age; not knowing the neurological outcome of infants). They were advanced-trained physiotherapists who completed basic and advanced training courses of GMA. From each video, the first observer randomly selected four full general movements and copied them onto a separate computer file for scoring. The next two main observers, based on those video clips, made the scoring criteria, and final GMs classified infant movements as normal FMs or absent FMs.

The third observer scored and classified GMs if the assessments of the main observers were not consistent.

The first videotaping of GMs was performed in the neonatal clinic just to gather a group of infants with potentially abnormal GMs. Infants included in the target study group were verified by a second GMA. Only infants with absent FMs at the second GMA were included in the study group.

#### 2.2.2. Experimental Procedure

**Figure 2:**
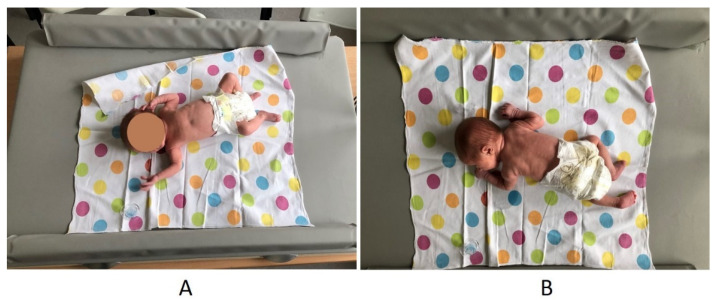
Posturometric test in supine (**A**) and prone (**B**) positions.

The PT was synchronized with the second GMA. On the force platform, the location of the infant’s head was marked using a mesh system. GMA and PT were started simultaneously due to special synchronizing software. A custom-built force plate with dedicated software and a video recorder connected to a computer with special software (a device designed and manufactured in the Department of Biomedical Electronics of the Institute of Electronics of the Silesian University of Technology in Gliwice) were used for the PT (Figure 2). The force plate included a large top plate with dimensions of 100 cm × 80 cm, four MEGATRON strain gauge transducer series KM 500 with a measuring range of 0–50 N [approximately 5 kG], and sensitivity of 2 m *v*/*v* (nonlinearity): 0.05% of the measuring range; hysteresis: 0.08% of the measuring range. PT was based on the analysis of the CoP trajectory and CoP area while the infant was lying in supine and prone positions on the force platform. The registration of posturographic parameters (Table 2) resulted from converting the changes in pressure forces on the transducers into changes in voltage values at their outputs, which were then amplified 1000 times by the AD623 instrumental amplifiers. The processing of the posturometric signal from the platform, the parameterization of the trajectory, and data archiving were carried out on the basis of a specially created computer program.

The infant was placed on the platform so that its belly button was in the middle of the platform, i.e., at the point of the intersection of the two diagonals (marked). Particular attention was given to maintaining the central position of the infant’s body on the force platform. The PT registration was carried out for 30 min, first in the supine position for 15 min and then in the prone position for 15 min. For analysis, we chose three recordings of 30 s each (i.e., between 4.30–5.00 min; 9.30–10 min, and 14.30–15.00 min). The mean value of these three measurements was used for further statistical analysis. The postural indices and formulas by which they were calculated are presented in Table 2. 

### 2.3. Statistical Analysis 

The software package SPSS v 26.0 (IBM Corp., Armonk, NY, USA) was used to carry out all statistical analyses. The sample size was calculated with G-power 3.1 software [35], and the required number of participants was 14. The normality of the quantitative variables was tested using Shapiro–Wilk’s tests (*p* > 0.05). Means (with standard deviations) were used to describe quantitative variables. Statistically significant differences between the tested and the control groups were identified using unpaired Student’s *t*-tests. In addition, a receiver operating characteristic (ROC) curve was applied to assess whether the new posturometric test data were able to discriminate between groups. The area under the curve (AUC) and the statistical significance of the ROC curve were described.

Because all the posturometric measurements were made in two positions of the infant (supine and prone), all analyses were performed separately for each position. All results were considered to be significant at the *p* < 0.05 level. 

## 3. Results

Participants were included in either the tested group (infants presented absent FMs) or the control group (infants presented normal FMs) based on the GMA. The inter-assessment agreement of stratification between absent FMs and normal FMs was perfect in both term and post-term GMA (ICC = 0.996–1.00 and ICC = 0.996–1.00; 95% confidence interval) (ICC = 0.986–1 and ICC = 0.985–1, respectively). 

Statistical analyses revealed significant differences between the infants from the tested group and the control group for all CoP parameters describing spontaneous CoP displacement in the supine position (Table 3). This was true for both types of posturometric indices, i.e., for those based on CoP shifts (SPL, VmaxCoP) and those based on the surface area of the CoP (ACoP, MCoPx, and MCoPy) (Table 3). Furthermore, the main postural parameter describing spontaneous sway of CoP (SPL) was twice as short in infants from the tested group compared with controls. Additionally, it was also observed that infants who presented absent FMs (tested group) showed a half smaller range of spontaneous CoP displacement, on average, in both the linear direction medial-lateral (MCoPx) and anterior-posterior (MCoPy) than those who presented with normal GMs (control group) in the supine position (Table 3). 

Although the differences between groups (tested vs. control) in the main postural control parameters describing spontaneous sway of CoP, such as SPL and VmaxCoP, in prone were not as great as in supine, they differed significantly (Table 4). However, no statistically significant differences were found between parameters describing the surface area of the CoP, such as ACoP, MCoPx, and MCoPy between infants with absent (study group) and normal (control group) FMs (Table 4). 

To determine the overall accuracy of the new posturometric test, the AUC values were used. AUC values greater than 0.9 were considered to be outstanding discrimination, those from 0.8 to 0.9 were excellent, and those from 0.7 to 0.8 were acceptable discrimination, while values less than 0.7 represented nonacceptable discrimination. A value of 0.5 means random discrimination [36]. 

The highest discrimination values between the tested group and the control group were shown for both CoP parameters describing spontaneous sway in the supine position, i.e., SPL and VmaxCoP (outstanding discrimination; AUC > 0.9). Nevertheless, the parameters describing the surface area of the CoP in the supine position (ACoP, MCoPx, and MCoPy) had an excellent discriminant value for the normal and absent FM patterns (AUC > 0.8) (Table 5). Considering the characterization of the postural control parameters in the prone position observed that the discriminant value for postural parameters describing spontaneous sway of CoP, such as SPL and VmaxCoP, was acceptable discrimination (AUC > 0.7), while it was nonacceptable and statistically insignificant for parameters describing the surface area of the CoP, such as ACoP, MCoPx, and MCoPy (AUC = 0.65) (Table 5). 

The best cut-off point (highest sensitivity while maintaining highest specificity) for individual variables with a significance test are presented in Table 5 and Figure 1. 

**Figure 3:**
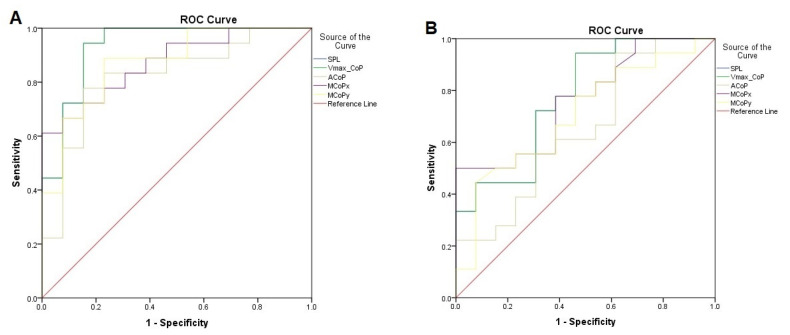
ROC curve and the optimal cutoff points for individual variables in supine (**A**) and prone position (**B**).

The ROC curves and the optimal cutoff point for individual postural parameters in two conditions of examination in supine (A) and prone (B) positions are presented in Figure 3. The diagonal line represents no discrimination, while the curves represent the sensitivity and specificity of individual postural parameters at different cutoff points. 

## 4. Discussion

The design and construct validity of the new PT for the recognition of postural control disturbances in pre-term, high-risk infants was determined by comparing its outcomes with the results of the assessment of general movements (GMA) for the early recognition of neurological deficits in pre-term infants.

The main finding of our study was the recognition of the correlation between abnormalities in postural control measured by new PT in pre-term infants with their absent FM patterns at 12–14 weeks post-term age. The comparison analysis between pre-term infants with absent FMs vs. pre-term infants with normal FMs in PT in a supine position presented altered postural control parameters in infants with absent FMs, such as (1) significantly shorter sway path length, (2) significantly slower velocity of CoP displacement, and (3) a significantly smaller range of spontaneous CoP displacement in both linear directions, i.e., medial-lateral and anterior-posterior in comparison with normally developing pre-term infants (controls). Differences between groups (tested vs. control) regarding the main postural control parameters describing spontaneous CoP sway and area in the prone position were not as evident as in supine.

To assess whether the new PT data were able to discriminate between infants who presented absent FMs (study group) from infants who presented normal FMs (control group) the Receiver Operating Characteristic (ROC) curve was applied. The outstanding discriminant capacity (AUC > 0.9) of both CoP parameters describing spontaneous sway in the supine position, i.e SPL and VmaxCoP, and excellent discriminant value of the parameters describing the surface area of the CoP in the supine (ACoP, MCoPx, and MCoPy) in examination in supine position between groups confirmed that new PT has the ability to discriminate between pre-term infants at risk for neuromotor deficits, i.e., those who presented absent FMs and normally developing pre-term infants, i.e., these who presented normal GMs.

The strength of this study is the use of the pressure-sensitive platform based on very sensitive transducers and equipped with a large tabletop, resembling a changing table for babies adapted for the safe examination of infants in horizontal positions. So far postural control in children has been assessed by means of posturography, i.e., measurement of spontaneous CoP displacement using a force platform. However, the force platforms (e.g., Kistler, AMTI) are limited in their clinical utility for posturometric assessment of infants because they are not sufficiently adapted to the specific anthropometric characteristics of infants in the supine position, such as small body size and very low body weight. 

Validation of CoP measured using a force platform in a horizontal (supine or prone) position has not been published thus far. Moreover, only a few studies have been concerned with the recognition of postural control of infants in the first months of life using CoP methodology [23,28,29,31,32]. 

Two studies by Dusing and co-workers assessed the displacements of CoP in the supine position at an early stage of infants’ development using a pressure-sensitive mapping method, but it was related to the comparison between pre-term and full-term infants [28,29]. There were recognized that premature infants exhibited more stereotypic patterns of movement, resulting in larger, but repetitive, CoP excursions than full-term infants. However, both examined populations (pre-term and full-term infants) as well approach to the CoP movement analysis in the above studies were different than ours. Therefore, these results cannot be directly compared. 

Probably, most similar to the presented study is the work of Støen and colleagues who analyzed supine infant movement relative to an observational clinical classification of high risk for CP according to GMs concept, where one of the measures was the standard deviation of the movement of the centroid over the duration of the recording [23]. Støen and colleagues have reported that infants with absent fidgety movements, i.e., infants at risk for motor impairment demonstrated greater variability in the centroid movement i.e., greater instability during movement [23]. The above study quantified the magnitude of the variability in postural control using root mean squared of the CoP displacement, while our study was based on spontaneous sway of CoP and velocity of the CoP displacement evaluation, so these results cannot be directly compared with our findings. 

Due to the lack of reference for the results of posturometric measurements of supine postural control in pre-term infants up to three months of age, the obtained results in our study are difficult to compare with those found in previous studies. This study is a feasibility study and reports pilot findings; therefore, it is difficult to draw definite conclusions. 

Nevertheless, the current results showed that the presence of abnormalities in postural control in preterm infants as measured by new PT correlates with their absent FMs pattern at 12–14 weeks post-term age. Based on these preliminary results, it can be concluded that the new PT in the supine position based on measurement of the CoP displacement has been demonstrated to be valid and can be a particularly revealing indicator for the development of postural control abnormalities in pre-term infants.

Further construct validity and reliability studies of presented PT are needed to provide some evidence that pre-term infants at risk for neuromotor deficits who present abnormal GMs and normally develop pre-term infants, i.e., these patients who presented with normal GMs differ in the nature of the development of postural control.

## 5. Study Limitations

This study has several limitations that must be considered and addressed in follow-up studies. First, due to the fact, that commonly used for CoP displacement measurements force plates (e.g., Kistler, AMTI) are not sufficiently adapted to the specific anthropometric characteristics of infants, such as body size and first of all very low body weight of the pre-term infant, the custom-built force plate used in our study has not been compared with state-of-the-art force platforms. Second, due to large differences in sampling frequency (our force plate 50 Hz vs. sensory mats only 5 Hz), it has not been compared with pressure-sensitive mats (PSM). Third, the number of participants in our study seemed relatively small; however, the low prevalence of CP (and thus abnormal GMs), which remains at 2–3 per 1000 live births, was a significant limitation in recruiting a greater number of infants with consistently presented abnormal GMs in the tested group for this study. 

## Figures and Tables

**Table 1:** Characteristics of the control group and tested group.

Parameter	Control Group (*n* = 18)		Tested Group (*n* = 19)	
	Mean (SD)	Min–Max	Mean (SD)	Min–Max
Birth weight (grams)	975.83 (273.41)	690–1850	949.68 (209.50)	650–1430
Gestational age (weeks)	27.72 (2.16)	24–32	27.15 (1.50)	25–30
Apgar at 5th minute (score)	6.33 (2.40)	1–9	5.31 (1.67)	3–8
Neonatal medical index—NMI (score)	2.1 (0.9)	1–5	3.6 (1.6)	1–5
Length of time stay in hospital (days)	27 (16)	2–65	31 (20)	5–85
Delivery; Normal, *n* (%) Caesarean, *n* (%)	8 (44) 10 (56)		6 (32) 13 (68)	
Sex; Girls, *n* (%) Boys, *n* (%)	6 (33) 12 (67)		6 (32) 13 (68)	

**Table 2:** The posturometric indices are based on CoP shifts, and the posturometric indices are based on the surface area of the CoP during lying [34].

Posturometric Indices Based on CoP Shifts during Lying	
Vmax CoP	Maximal velocity of the CoP displacement [cm/s]
SPL	Sway path length of the CoP [cm]
**Posturometric indices, based on the area of the CoP during lying**	
ACoP	area of CoP shifts under the unrolled trajectory [cm^2^] $ACoP=\overset{N}{\underset{i=2}{\sum }}p\left(i\right)$ where: $p(i)=\sqrt{ob\left(i\right)\cdot \left[ob\left(i\right)-r\left(i-1\right)\right]\cdot \left[ob\left(i\right)-r\left(i\right)\right]\cdot \left[ob\left(i\right)-l\left(i\right)\right]}$ *p*(*i*) is the surface area of a triangle comprised of two successive points of a given trajectory (T_c_(*i*−1), T_c_(*i*)), and the point T_c0_ represents the center of that trajectory; *l*(*i*) is given by the formula, whereas values of *r*(*i*), *r*(*i*−1), and *ob* (*i*) are calculated using the following equations: $r\left(i-1\right)=\sqrt{{\left[x_{c}\left(i-1\right)-x_{c0}\right]}^{2}+{\left[y_{c}\left(i-1\right)-y_{c0}\right]}^{2}}$ $ob\left(i\right)=\frac{l\left(i\right)+r\left(i\right)+r\left(i-1\right)}{2}$
MCoPx	Mean medial-lateral linear displacement of the CoP [mm] $MCoPx=\frac{\sum_{i=1}^{N}\left|x_{C}\left(i\right)-X_{CO}\right|}{N}$
MCoPy	Mean posterior-anterior displacement of the CoP [mm] $MCoPy=\frac{\sum_{i=1}^{N}\left|y_{C}\left(i\right)-Y_{CO}\right|}{N}$

**Table 3:** Main center-of-pressure (CoP) parameters describing spontaneous sway of CoP in the supine position in the control group (infants presented normal fidgety movements) vs. the tested group (infants presented absent fidgety movements).

PARAMETER	Control Group (*n* = 18)	Tested Group (*n* = 19)	Mean Difference	Statistical Test
	M (SD)	M (SD)	(95% CI)	*p*-Value
SPL	358,33.61 (12,702.11)	157.38 (83.37)	200.94 (118.27–283.62)	<0.001
VmaxCoP	11.94 (4.23)	5.24 (2.77)	6.69 (3.94–9.45)	<0.001
ACoP	245.19 (106.28)	144.71 (68.50)	10.04 (3.15–16.93)	0.006
MCoPx	7.94 (3.45)	3.37 (1.81)	4.57 (2.61–6.53)	<0.001
MCoPy	7.63 (3.44)	3.37 (1.95)	4.25 (2.08–6.42)	<0.001

M, mean; SD, standard deviation; CI, confidence interval; Student’s *t*-test, *p*-value.

**Table 4:** Main center-of-pressure (CoP) parameters describing spontaneous sway of CoP in the prone position in the control group (infants presented normal fidgety movements) vs. the tested group (infants presented absent fidgety movements).

PARAMETER	Control Group (*n* = 18)	Tested Group (*n* = 19)	Mean Difference	Statistical Test
	M (SD)	M (SD)	(95% CI)	*p*-Value
SPL	391.64 (144.29)	245.49 (129.53)	146.15 (43.14–249.16)	0.007
VmaxCoP	13.05 (4.80)	8.18 (4.31)	4.87 (1.43–8.30)	0.007
ACoP	32.25 (10.89)	25.11(13.26)	7.13 (−1.74–16.02)	0.111
MCoPx	8.95 (4.45)	5.36 (2.98)	3.59 (0.68–6.50)	0.017
MCoPy	7.56 (2.80)	5.74 (2.47)	1.82 (−0.17–3.80)	0.071

M, mean; SD, standard deviation; CI, confidence interval; Student’s *t*-test, *p*-value.

**Table 5:** The highest discrimination value between the tested group and the control group for individual parameters describing spontaneous sway of CoP in the supine and prone positions.

PARAMETER	Supine Position					Prone Position				
	HDV	SE	SP	AUC	95% CI	HDV	SE	SP	AUC	95% CI
SPL	212.42	94%	85%	0.932	0.836–1.00	298.39	72%	69%	0.774	0.604–0.943
VmaxCoP	7.08	94%	85%	0.932	0.836–1.00	9.94	94%	85%	0.774	0.604–0.943
ACoP	17.22	83%	77%	0.821	0.665–0.976	-	-	-	-	-
MCoPx	4.52	78%	77%	0.872	0.751–0.992	6.13	78%	62%	0.763	0.596–0.930
MCoPy	4.38	89%	78%	0.872	0.746–0.998	-	-	-	-	-

HDV, highest discrimination value; SE, sensitivity; SP, specificity; AUC, area under the curve; CI, confidence interval.

## Data Availability

The data supporting the results of this study are available from the corresponding author upon reasonable request from any qualified investigator.
